# Scope of Onsite, Portable Prevention Diagnostic Strategies for *Alternaria* Infections in Medicinal Plants

**DOI:** 10.3390/bios13070701

**Published:** 2023-07-01

**Authors:** Sadhana Shukla, Pushplata Singh, Shruti Shukla, Sajad Ali, Nidhi Didwania

**Affiliations:** 1 Manav Rachna Centre for Medicinal Plant Pathology, Manav Rachna International Institute of Research and Studies, Faridabad 121004, India; sadhana.shukla@teri.res.in; 2 TERI-Deakin Nanobiotechnology Centre, The Energy and Resources Institute, Gurgaon 122003, India; pushplata.singh@teri.res.in (P.S.);; 3 Department of Biotechnology, Yeungnam University, Gyeongsan 38541, Republic of Korea

**Keywords:** medicinal plant, *Alternaria*, pathogen detection, nanotechnologies, portable biosensors

## Abstract

Medicinal plants are constantly challenged by different biotic inconveniences, which not only cause yield and economic losses but also affect the quality of products derived from them. Among them, *Alternaria* pathogens are one of the harmful fungal pathogens in medicinal plants across the globe. Therefore, a fast and accurate detection method in the early stage is needed to avoid significant economic losses. Although traditional methods are available to detect *Alternaria*, they are more time-consuming and costly and need good expertise. Nevertheless, numerous biochemical- and molecular-based techniques are available for the detection of plant diseases, but their efficacy is constrained by differences in their accuracy, specificity, sensitivity, dependability, and speed in addition to being unsuitable for direct on-field studies. Considering the effect of *Alternaria* on medicinal plants, the development of novel and early detection measures is required to detect causal *Alternaria* species accurately, sensitively, and rapidly that can be further applied in fields to speed up the advancement process in detection strategies. In this regard, nanotechnology can be employed to develop portable biosensors suitable for early and correct pathogenic disease detection on the field. It also provides an efficient future scope to convert innovative nanoparticle-derived fabricated biomolecules and biosensor approaches in the diagnostics of disease-causing pathogens in important medicinal plants. In this review, we summarize the traditional methods, including immunological and molecular methods, utilized in plant-disease diagnostics. We also brief advanced automobile and efficient sensing technologies for diagnostics. Here we are proposing an idea with a focus on the development of electrochemical and/or colorimetric properties–based nano-biosensors that could be useful in the early detection of *Alternaria* and other plant pathogens in important medicinal plants. In addition, we discuss challenges faced during the fabrication of biosensors and new capabilities of the technology that provide information regarding disease management strategies.

## 1. Introduction

Medicinal plants have always been important and a matter of concern for people as they had therapeutic-based usage in the past [1]. In general, medicinal plants have been a healing resource in communities around the world for thousands of years. Nevertheless, they remain important today as primary health care for about 85% of the world’s population [2] and as a resource for pharmaceutical research, from which 80% of all synthetic drugs are derived [3]. Medicinal plants were used as traditional remedies to treat a number of lethal diseases from ancient times and produce diverse chemical scaffolds that can interact with biological targets. India is the place of origin of a wide variety of medicinal plants and precious traditional medicine knowledge [4]. It is reported that 80% of the population in developing countries benefit from herbal products for therapeutic uses and purposes.

**Figure 1:**
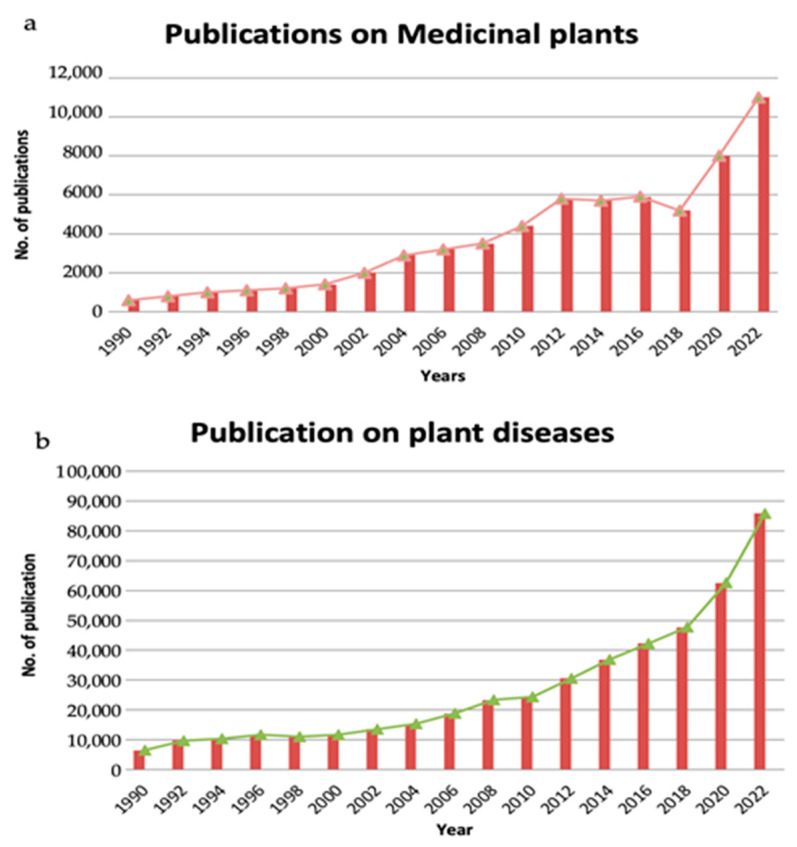
(**a**) Number of papers published in the past 32 years on medicinal plants and related to their evolutionary research in these published articles till the years 2020 referred from Salmerón-Manzano et al. (2020) [6]. (**b**) Highlights of data on the number of studies published on diseases in plants with the keyword “plant disease” in different databases (ScienceDirect, Nature).

Like other crop plants, medicinal plants are constantly challenged by an array of biotic inconveniences that pose a serious threat to their survival. Among them, fungal pathogens such as *Alternaria* spp. cause severe economic losses in medicinal plants and affect their product quality [4]. The co-occurrence of abiotic and biotic stresses in medicinal plants has increased dramatically as a result of global climate change, exposing the risk of their existence. Commercial cultivation has brought the unavoidable problem of more pests and diseases, ultimately leading to crop loss at various levels. Additionally, overharvesting has placed many medicinal species at risk of extinction [5]. Therefore, *Alternaria* diseases could further increase the risk of their extinction. Hence, there is a need to develop accurate, sensitive, and rapid detection tools to detect *Alternaria* diseases at early stages under both lab and field conditions. Several researchers have published data on medicinal plants in terms of therapeutic values, value chain losses, and commercial aspects; however, there is still a lack of big data values of medicinal plants related to their loss due to limited disease diagnostics. Figure 1a illustrates 32 years of data on the number of published articles on medicinal-plant-based evolutionary research. Similarly, Figure 1b represents data on the number of studies published on plant diseases with the keyword “plant disease” in different database systems (ScienceDirect, Nature) pertaining to medicinal plants, which gives clear indications of the need for advanced research on medicinal plants’ diagnostic systems.

*Alternaria* is a cosmopolitan genus of fungi that are saprophytic, endophytic, and pathogenic to plants and animals and is ever present in many favorable and anthropogenic environments [7]. Several edible medicinal herbs have been utilized for more than thousands of years to prevent and treat human disorders. In addition, these herbs are consumed as spices, additives, and/or edible foods. Unfortunately, these medicinal herbs can become contaminated with *Alternaria* toxins (ATs) before harvest, raising safety concerns [8]. Among *Alternaria* pathogens, *Alternaria alternata* has been reported to cause leaf spot and leaf blight diseases in various medicinal plants, and its toxins are of great concern [4]. Therefore, pathogens in medicinal plants and their negative impact should be identified as soon as possible to have more surety and take cost-effective measures, which play a crucial role in ensuring the security of food products and reducing yield loss [9]. 

Nowadays, pathogen-related losses of medicinal plants are responsible for the reduction in the country’s financial returns [10]. Therefore, the earliest diagnostics with the properties of rapidness, inexpensiveness, and accuracy have become the best combinations in plant pathological studies. Furthermore, in a global market, innovative disease-sensitive and field-ready tools represent a new frontier from a diagnostic laboratory perspective, ensuring that the instruments and tools used are much more suitable in case of operational situations [11]. The commonly established methods, such as polymerase chain reaction [12], fluorescence in situ hybridization [13], enzyme-linked immunosorbent assay [14], immunofluorescence [15], flow cytometry [16], and gas chromatography–mass spectrometry [17], are already existing and broadly used for disease identification in plants. However, they are comparatively tough to use, require experts, are tedious, and are unsuitable for on-field diagnostic and early management strategies because they cannot provide real-time detection data [18]. Meanwhile, some imaging techniques, for example, thermography and fluorescence imaging, are capable of on-field disease detection and analysis, but they are sensitive to variable changes in the environment and lack specificity for types of diseases [19]. 

A previous study reported the role of various biosensors in pathogenic disease detection in plants, which opens new directions for the development of a new pathogen-specific detection tool kit [17]. Recently, advancements in nanotechnology have paved the way for the better development of nanoparticle-based easy assays for the detection of bioanalytes, which emerged in the improvement of much-sensitive biosensors because of new nanofabrication methods. Nanoparticles (NPs) with uncommon optical properties and more surface area are widely used for the easy detection of bioanalytes of interest in multiple samples [20]. The great physicochemical properties of NPs have proved them to be much more suitable over conventional detection techniques for diagnostic purposes; emerged as a potential way to improve the sensitivity, accuracy rate, and fastness of plant pathogen identification tools; and paved the way for high-throughput analysis [21], which might be suitable for the detection of *Alternaria* diseases. 

The specificity of nanomaterial-based biosensors could be efficiently improved due to the use of enzymes, antibodies, DNA, and bacteriophage [17]. The present review is an update on the current global scenario of medicinal plant losses due to *Alternaria* infections and further elaborates on the possible diagnostics for early, rapid, and cost-effective detection via nanomaterial-based biosensing strategies.

## 2. Global Status of Disease Burden on Medicinal Plants

Contamination of food and crops by environmental contaminants, synthetic drugs, residues of chemicals, and pathogens, including various other anthropogenic and naturally present contaminants, is a serious problem in public health worldwide [22,23]. In particular, fungal disease has been a major constraint in medicinal plants across the globe [7]. In this review, we summarize different fungal diseases in medicinal plants with the main focus on *Alternaria* pathogens (Table 1). 

*Alternaria* toxins (ATs) are a serious concern to humans and animals owing to their wide range of side effects. Data on the occurrence of ATs in medicinal herbs are relatively limited, as previous studies did not routinely examine the amounts of these toxins in herbs and edible products [43,44]. ATs have recently been called “emerging mycotoxins” of medicinal plants. Despite this, there are recently no monitoring guidelines and limitations for ATs in food and/or medicinal plant products. Therefore, there is an urgent need to find reliable analytical methods to monitor ATs that are also applicable to herbs [18,37]. 

### Alternaria Toxins in Medicinal Plants and Their Effects on Humans

*Alternaria* is capable of producing several secondary metabolites with toxicity under specific conditions, also called ATs [45,46]. *A. alternata* and *A. tenuissima* were found to be associated with muskmelon foliar diseases in China in the area of Beijing [47]. These diseases significantly reduced the production and quality of important crops, vegetables, and fruits, resulting in negative impacts on the agricultural economy [22]. *Alternaria* species range from saprophytic to pathogenic fungi, and therefore, they are able to cause fungal diseases in medicinal plants, such as withering, leaf spot, plant decay, and other visible symptoms [48,49]. 

**Figure 2:**
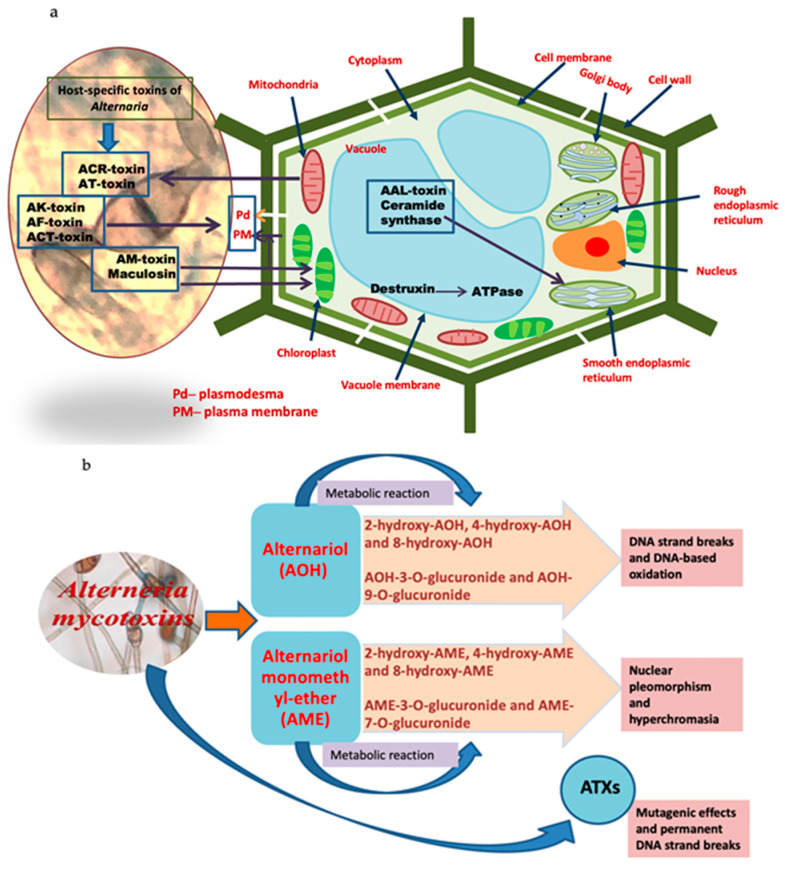
*Alternaria* toxins and their targets: (**a**) presenting the target sites of HSTs produced by *Alternaria* species in the plant cells; (**b**) representing enzymatic metabolism for the development of *Alternaria*-developed toxicants and *Alternaria* major mycotoxins.

*Alternaria* species produce various host-specific (HSTs) and non-host-specific (nHSTs) phytotoxins, which affect different plant cellular organelles. Reported non-host-specific toxins include “tentoxin (TEN), alternaric acid, alternariol (AOH), alternariol 9-monomethyl ether (AME), brefeldin A (dehydro-), alternuene (ALT), altertoxin-I, Altertoxin-II, altertoxin-III, zinniol, tenuazonic acid [TeA], curvularin, and alterotoxin (ATX) I, II, III” [28,50]. On the other hand, HST toxins are “Japanese-pear-pathotype (A. kikuchiana) (AK), Strawberry-pathotype (AF), Tangerine-pathotype (A. citri) (ACT), and Apple pathotype (A. mali) (AM), including, *Alternaria alternata* lycopersici toxins (AAL) and ACR-toxins, such as maculosin and destruxin A and B, etc.” [22]. Further, we have shown the effect of different ATs on plant cellular organelles, as shown in Figure 2a. Non-host-specific toxins act as contaminants in fruits and vegetables [51]. Figure 2b deals with different enzymatic metabolisms in *Alternaria* for developing *Alternaria* toxicants, including mycotoxins, namely AOH, AME, and ATXs, produced by *Alternaria*, which are hazardous to humans with different cytotoxic and genotoxic effects. With their pathogenic properties, they affect many agricultural products and may then get into the food chain effectively [52,53]. In 2011, the Food Safety Authority (EFSA) worked on assessing the risk of *Alternaria* toxins, and found that these toxins could induce genotoxicity and cytotoxicity, including reproductive and developmental toxicity [23]. 

Cytotoxicity could inhibit cellular growth [54], restrain protein synthesis, and induce apoptosis [55]. It is mentioned in previous reports that the high incidence of esophageal cancer might be due to the ingestion of *Alternaria*-mycotoxin-contaminated food, recorded in the Linxian area of Henan Province, China [56]. Recent studies have shown that *Alternaria* mycotoxins exhibit genotoxic effects, and a few of them could induce gene locus mutation. DNA damage or synthesis disorder, chromosome aberration, and other effects of *Alternaria* mycotoxins have also been reported through in vitro studies [22].

## 3. Preventive Measures for *Alternaria* Infections in Medicinal Plants

*Alternaria* diseases in medicinal plants are a major concern as they can considerably impact the economy by drastically reducing their yield and quality. The conventional approaches to controlling and preventing *Alternaria* infections may include the spraying of chemically synthesized fungicides; for example, the fungicides pyraclostrobin and azoxystrobin in combination with difenoconazole were generally effective in cases of leaf blight disease [57,58]. In addition, a few medicinal plants have been protected from diseases by integrating resistant genotypes of Jerusalem artichoke with *Trichoderma harzianum* isolate to prevent *Alternaria* leaf spot [42,59]. As a research advancement, researchers have used various other biocontrol strategies for fungal disease control, including liquid copper fungicides, powdered sulfur fungicides, and potential bacterial strains of *Bacillus subtilis*, as well as other physical control methods, such as removing dead plant matter, drip irrigation, planting disease-free seeds and resistant cultivars, sterilized tools, and rotation of crops [60,61]. Although chemical treatment remains the most important method for reducing the incidence of fungal disease, frequent use can make fungal populations less sensitive. Moreover, excessive spraying can lead to environmental pollution and impose a large economic burden on producers [62]. However, a few methods, such as mulching, crop rotation, cleaning up fallen leaves, and elimination of water on leaves, can help the slow spread of *Alternaria* spores in the soil and control *Alternaria* diseases in plants [63]. Due to the above limitations, it is suggested that optical sensing techniques can help to identify the primary diseases and areas of varying disease severities in the field, especially in the case of *Alternaria* spp. [64]. To prevent and control the spread of pathogenic disease, producers need to be able to identify causative strains accurately, sensitively, and quickly. This allows you to select and implement the best possible disease management strategies [58].

## 4. Common Available Diagnostic Strategies

Many fungal pathogens during disease development can impose similar effects on medicinal plants, so it becomes crucial to differentiate between causal fungal pathogenic species to ensure well-informed management [65]. In the field, biosensors are preferred for detecting lethal pathogens in plants because they are inexpensive, require much less expertise, and identify target pathogens very quickly with high specificity and sensitivity [66,67].

### 4.1. Conventional Methods

Conventional methods are feasible [68] for a person without any strong science background as the steps are easy to understand. They are reliable and have easy steps to develop the protocol but are very tedious and unsuitable for the onsite early detection of *Alternaria* infections and viruses or bacteria that colonize inside the plant tissues [69].

Some of the conventional disease diagnostic methods in developing countries are mentioned below.

#### 4.1.1. Visual Observation

Depending on the symptoms, visual monitoring can be performed in the plant samples; it is frequently feasible to detect the primary pathogenesis of a worm, fungal spp., bacterial spp., or viruses [70]. Plant diseases can be identified by examining leaf curling, color change, and spotting of either brown or white color [71]. The entire procedure occasionally takes so long that the disease cannot be completely eradicated. Large areas also make this process exceedingly laborious [72].

#### 4.1.2. Microscopy

Highly precise microscopes are needed and are often used tools for precise diagnostics. The contaminated plant portions are collected for examination via a microscope after being observed visually in the lab [73]. It is frequently feasible to make a diagnosis by examining the hyphae, microsclerosis, conidiophores, conidia, and bacterial cell clusters of a pathogenic fungus on the diseased plant components under a microscope [74]. However, in unfavorable weather, a fungus might not even form spores or have fungal mycelium on the affected surface [75].

#### 4.1.3. Mycological Diagnosis

Researchers can record the proliferation and pattern of pathogens using the moist chamber method [76]. The “wet chamber” technique includes incubating a portion of the ill plant under a high-humidity maintained chamber (Petri dishes, etc.). In this method, fungi in the diseased tissues proliferate and start to manifest as a result of favorable conditions [77]. Under a commonly used microscope, it is possible to identify the genera and species of pathogenic fungal organisms through the observation of their hyphae, macro conidia, and microconidia [78]. Certain phytopathogens may be difficult to identify using moist chamber techniques [79].

#### 4.1.4. Biological Assays or Indicator Plant Tests

This methodology is commonly used for the diagnosis of phytoviruses and phytoplasmas, in addition to other diagnostic methodologies. Biologically based diagnostics are implied on indicator trees and herbaceous plants, as a testing [80]. Infection is transmitted to indicator plants by mechanical inoculation or injection. Symptomatic leaves rubbed onto healthy indicator plants obtain and are injected with standard buffer suspensions [80]. The artificial infection method by micrografting is also used for indicator trees and shrubs, and such tests are performed under strictly isolated and controlled conditions. Its main drawbacks are long latency and the need for culture chambers, isolated from others [81].

### 4.2. Rapid Lab-Based Diagnostic Methods

#### 4.2.1. Antigen-/Antibody-Based Diagnostics 

Another molecular approach for diagnosing plant diseases with the use of antibodies and color-changing assays is the enzyme-linked immunosorbent assay (ELISA) [14]. With this technique, antibodies attached to enzymes are used to precisely bind the specific epitopes (antigens) from viruses, bacteria, and fungi. ELISA is the most advanced serology-derived diagnostic technique for fungi [77]. Based on color changes brought on by the reaction of the substrate and the immobilized enzyme, the detection can be seen [49]. The techniques of monoclonal antibodies and recombinant antibodies can significantly increase the performance of ELISA and are capable of identifying tiny molecules, viruses, and their quantitative markers [79]. Although there are many methods for immunodiagnostics, among them, ELISA is considered sensitive and accurate, allowing a definitive diagnosis [75]. The drawback of the ELISA technique is that it has low sensitivity for complex bacteria [82].

#### 4.2.2. Molecular Genetic Identification

Numerous techniques, including “nucleic-acid-hybridization, polymerase-chain-reaction (PCR), reverse-transcription PCR (RT-PCR), real-time PCR, and DNA-derived-microchips”, are available for the molecular genetic characterization of harmful microorganisms in plants [83]. Plant pathogen detection now makes extensive use of PCR techniques as they are greatly sensitive and advanced, such as RT-PCR, which has been used for lethal plant pathogen identification [12]. The idea behind multiplex PCR is to perform a single reaction while simultaneously detecting many types of DNA or RNA particles and a speedy identification of plant diseases using real-time PCR technologies [84]. Pathogen diagnostic applications have adopted a variety of PCR amplification assay types to enhance the assay specificity, for example, nested PCR; signal identification, for example, magnetic capture hybridization and ELISA PCR; and multiplexing ability as multiplex PCR techniques [85]. A single picogram of pure fungal DNA can be identified using simple, dependable, and scalable PCR techniques. Nielsen and colleagues (2002) found the increasing detection sensitivity of quantitative PCR (qPCR), using real-time monitoring of amplification, depending on fluorescence to precisely establish the starting concentrations [62].

Although the PCR approach has been utilized for multiplexed pathogen detection, still it is constrained by the lack of operational flexibility even though it enhances the sensitivity of detection due to the fidelity of DNA amplification [46]. For PCR-based identification, nucleic acid isolation, genetic marker selection, specific primer designing, PCR machine, electrophoresis, DNA purification, and result determination are all required [84]. Inhibitors in the sample, polymerase activity, PCR buffer, and deoxynucleoside triphosphate concentration all effect PCR performance. This is the limitation of this technique in practical applicability for disease field sampling [62,86]. 

#### 4.2.3. Flow Cytometry (FCM) 

Flow cytometry (FCM) is a commonly used laser-beam-based optical technology for cell number counting and sorting, finding biomarkers, and creating proteins [87]. FCM is used to quickly identify cells as they go fast on an electronic detection device in a liquid stream. The capacity to test multiple parameters simultaneously is a benefit of this technology. The method [88] makes use of an incident laser beam and evaluates the scattering and fluorescence of the beam reflected off the sample. Although FCM has mostly been used to research cell cycle kinetics and antibiotic sensitivity, it has also been used to count live and dead bacteria, and it characterizes bacterial DNA and fungal spores [16]. Detection with this technique is based on nonspecific staining of DNA or characterization of scattered patterns and autofluorescence [89].

#### 4.2.4. Gas Chromatography 

It is a non-optical-based indirect-type method for detecting lethal plant disease, including volatile chemical character profiling of infected plants. Volatile organic compounds (VOCs) are produced in green-leafed plants [90] and emitted differently from plants infected by diseases and mechanical damage [91]. The type and form of infection might be determined through the profiling of such VOCs, which could then be used for illness diagnosis and confirmation [92]. The particular VOC that is irrelated to disease particularly could be determined by gas chromatography [GC] analysis of the volatile signature of plants [93]. 

Gas chromatography and mass spectrometry are frequently coupled (GC–MS) to improve the performance of compound differentiation and to identify unidentified compounds in volatile samples [94]. However, the requirement for a sample precollected VOC for a longer length of time prior to data processing substantially restricts the application of GC/GC–MS on the field, unlike the imaging technology, by which the data can be obtained onsite directly [17].

Therefore, generally used traditional lab strategies for plant pathogen detection could not be applied for the detection of *Alternaria* infections because they are time-consuming and have multiple drawbacks, in addition to the fact that they are unsuitable for the variables and can cause severe loss due to time limitations and incorrect diagnostics.

## 5. Portable Diagnosis Techniques

The early detection of plant pathogens is an appropriate preventive strategy for the management of crop yield and quality. For this reason, effective diagnostic techniques and tools, which are simple, specific, rapid, and economical, are needed to be developed [95].

### 5.1. PCR-Based Systems

Whatman FTA cards developed by GE Healthcare are a fast, cost-effective, and simple method for extracting DNA from different plant tissues and can store DNA at room temperature for years, which can be used later for PCR purposes [96]. To diagnose a disease directly in the field easily, portable devices, such as “Palm PCR”, were developed by the “Ahram Biosystems” Korea, which has helped to overcome barriers of electrical supply [97]. Additionally, a “Twista recombinase polymerase amplification (RPA) fluorometer” developed by TwistDx was utilized for immediate and fast diagnosis, allowing an on-time suitable treatment [98]. This technology was used for the detection of *Phytophthora* spp. in plant tissue [99]. Despite these advantages, portable PCR devices still face challenges, such as sample preparation, DNA extraction, accurate temperature control systems, and sample evaporation in open devices [100]. 

### 5.2. Lateral Flow Assays (LFAs) 

LFAs based on immunodetection and immunoprinting kits are considered among the first grower-friendly pathogen monitoring methods [101]. For the detection of plant diseases, tissue-printing ELISA and lateral-flow devices have been manufactured to allow onsite detection [17]. To date, LFA devices are available in the market for the detection of several plant pathogens, such as *Phytophthora* spp., *Ralstonia solanacearum*, and potato virus, with easy handling and steps (e.g., pocketdiagnostic.com); hence, they are very handy for farmers. However, LFAs have some disadvantages, such as limited potential for multiplex detection of target analytes. Additionally, low sensitivity has been reported for some LFA-based biosensors [102]. 

### 5.3. Microsphere Immunoassays (MIA)

MIA techniques have emerged as a promising alternative for microbial detection. This technique is based on microspheres coded with different fluorophores. Briefly, the microspheres are conjugated with specific antibodies to capture the pathogen antigen, and a magnet is used to separate the bound and nonbound microspheres [103,104]. After the separation, specific antibodies linked with a reporter fluorophore are added to the microsphere sample mix. A Luminex xMAP analyzer excites the internal dyes of the microspheres with a red laser and the reporter fluorochrome, captured by the antigen, with a green laser, allowing the specific detection of multiple analytes at the same time [100].

### 5.4. Hyperspectral Imaging Techniques 

These techniques can be utilized between 350 and 2500 nm hyperspectral imaging to gather important data regarding the health of plants [105,106]. The hyperspectral examination combines optical spectroscopy and picture investigation strategies, permitting both physiological and morphological parameters to be assessed simultaneously [107]. The hyperspectral imaging technique (HIS) has been applied for the early detection of citrus and apple fruit diseases caused by pathogens in the field and has also been used to estimate the ripeness of strawberries in the field [108]. In any case, each interaction between a plant and a pathogen has a certain spatial and worldly flow, and these forms influence a diverse range of the electromagnetic parameters [106]. However, HIS has still not been utilized for the early detection of black spots [109].

### 5.5. Fluorescence Imaging 

This method involves measuring the fluorescence that came off from the chlorophyll on the leaves by calculating the reaction of the incident light. Based on changes in the photosynthetic instrument and electron transport reactions [110], the fluorescence patterns can be used to analyze the level of pathogen infections. Efficient sensors along with LED [111] are frequently used in chlorophyll fluorescence imaging equipment to measure photosynthetic electron transport [112]. The variations in photosynthetic activity brought on by biotic and abiotic inconvenience over the leaf area have been studied using this method [19]. The distinction and measurement of fungi infections have been demonstrated to benefit from the integration of fluorescence imaging and image analysis tools [113]. It is challenging to use existing chlorophyll fluorescence imaging technologies in typical agricultural greenhouses or field conditions because the preparation of the plants must adhere to a tight routine [64]. In order to assess plant disease at the field level, research has focused on observing fluorescence aspects from sunlight-induced reflectance at the field level and using chlorophyll-fluorescence-based imaging and thermography to understand and compare infections by a fungus and a virus in plant leaves [82].

### 5.6. Loop-Mediated Isothermal Amplification (LAMP)

LAMP assay is a single-step method in which 4 to 6 primers bind the target sites laterally using a strand displacement *Bst* DNA polymerase, allowing amplification under isothermal conditions [114,115], as well as gel electrophoresis, turbidimeter, lateral flow dipstick, or the naked eye used for detecting amplified products [116]. LAMP assay was used for the rapid detection of *Alternaria* spp., including *A. alternata*, on infected pears [3,117]. LAMP can be applied in the field and in laboratories. LAMP assay has some limitations and defects, as it contaminates easily, needs a source of light, and requires some skills for assessing the results [62]. 

## 6. Smartphone-Based Portable Device Diagnoses

Every plant releases volatile organic compounds (VOCs) as they breathe through the pores in their leaves. However, when a plant is diseased, the type and concentration of these VOCs change. Smartphone-based portable technologies can detect VOCs released by almost every plant within a fraction of a minute. Scientists at North Carolina State University developed a handheld device that is designed to be attached to the farmer’s smartphone, sitting over the top of its camera, and it works by sampling the airborne VOCs that plants release [118].

A WeChat applet-based mobile solution was developed for citrus disease diagnosis with a simplified DenseNet deep learning architecture, which was further connected to a server where web requests can be made and help to recognize diseases from the captured leaf or fruit image samples [109]. On the same basis, as a proof of concept, server and mobile client cloud-based applications using a ResNet50 deep learning architecture were reported for tomato disease classification [119]. Similarly, an in-field, automatic wheat disease diagnosis system along with a server for the disease identification steps and a mobile client for in-field image capturing was built [120]. However, these methods require internet connectivity and high computational power to process the images in a central server. Conversely, a mobile-based solution that can operate directly on mobile devices without any need of an internet connection was proposed and developed [121,122]. 

## 7. Nanomaterial-Based Biosensing Strategies for Plant Pathogen Detection

Nanomaterials are ideal candidates for the analysis of plant pathogens as their dimensions typically fall in the range of 1 to 100 nm, which can provide enhanced surface-to-volume ratio and unique chemical, optical, and electrical properties, which are not observed in the bulk counterparts [123]. Nanomaterials are normally synthesized via specific self-assembly procedures at the nanoscale level [124]. 

Nanoscale materials can also interact with biomolecular targets more efficiently due to their small sizes and fast diffusion rates [123]. Nanomaterials, such as carbon-based materials, such as graphene and carbon nanotubes, including graphite, silver (Ag), gold (Au), silica (Si), and copper (Cu) nanoparticles [125], can be prepared in many different morphologies ranging from spherical particles, cubes, rods, wires, plates, prisms, and core–shell structures to more complicated 3D architectures. In addition, new advanced nanomaterials, such as nanotubes, nanorods, nanospheres, nanoreefs, nanoclusters, tetrapods, nanopores, and nanochannels, have been created as a result of the growth of nanotechnology as sensing tools [126], which have been widely applied in the areas of in vivo imaging [127,128,129]. These could undergo shape transformation or agglomeration, which alters their chemical or physical properties in response to various external stimuli [130]. 

Biosensor devices are derived by their bioinspired receptor units with differently efficient specificities to their targeted analytes [131]. For example, enzymes can react with certain molecules rapidly and selectively, and nucleic acids can bind to their complementary sequences delicately on the nanoscale [132]. The biorecognition components may consist of enzymes, nucleic acids, entire cells, aptamers, or polymers, whereas the analyte may be an ion, molecule, cell, nucleic acid, or protein [133]. The addition of nanomaterials (NMs) into a biosensor can detect the signals and provide a more accurate and specific identification of an analyte [134,135]. For example, carbon nanomaterials are suitable for the conjugation of biomolecules (enzymes, antibodies, DNA, cell, etc.) [132]. In addition, biomolecules can be immobilized and conjugated with other materials by surface modification through the recombination or introduction of chemical linkers [136]. In biotechnology-derived sensing techniques, gold nanoparticles (AuNPs) are commonly used; they can be used as carriers in the case of biorecognition molecules, antibodies, or aptamers, and can also be the labels for signal transduction and amplification [137]. For example, absorption-based colorimetric sensing involves aggregation-induced interparticle surface plasmon coupling of AuNPs, which results in a visible color change from red to blue. This concept has provided a practical platform for the detection of any target analyte that triggers the AuNPs’ aggregation or redispersion [138]. AuNPs are widely utilized in electrochemical-based biosensors as they can increase the surface area and conductivity and have been reported in the detection of microbial food pathogens [139].

These advanced nanomaterials have recently begun to play a crucial role in the creation of biosensors, tissue engineering, battery materials, and multifunctional materials [127,140]. The three main parts of a biosensor are (i) a biorecognition element for detecting the presence and concentration of an analyte; (ii) an optical, electrochemical, piezoelectric, or thermally based transducer surface; and (iii) a detector to measure the signals produced [141].

The sensing applications would be a fruitful strategy for developing a novel tool of diagnostics, especially for *Alternaria*. 

### 7.1. Colorimetric Sensing

**Figure 3:**
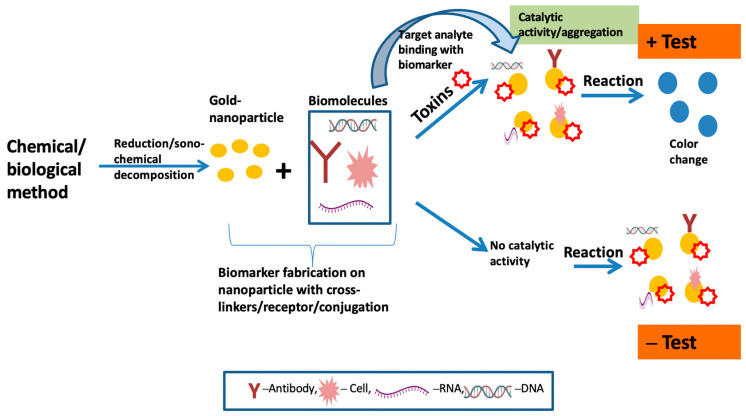
Schematic diagram of colorimetric-based biosensor for analyte detection.

Colorimetric sensors are based on simple chemical reaction principles and have become more usable and widespread due to their vast availability, simple use, inexpensiveness, and ability to respond sensitively and selectively to a variety of analytes. Colorimetric sensors are one of the types of optical sensors; they change color in response to external inputs if any modification to the physical or chemical surroundings qualifies as such a stimulus [142]. Four examples of colorimetric techniques include “indirect target-mediated aggregation, chromogenic substrate-mediated catalytic activity, point-of-care testing (POCT) devices, and machine learning-assisted colorimetric sensor arrays”, which can be utilized for pathogen analysis [143,144]. Figure 3 depicts a pictorial general working overview of colorimetric-based biosensors with the fabrication of AuNPs to identify the target analytes in samples. To attain mobility and intellectualization in clinical applications, colorimetric-sensor-based gold nanoparticles can be further integrated with newly developed portable devices, such as paper-based chips. This can be applied to smartphone readout applications and can help with the visualization and in situ detection of germs [69]. 

### 7.2. Paper-Based Sensing

Paper is a versatile material and gaining attention as a substrate for developing paper-based analytical devices (PADs) in point-of-care (POC) format, being an alternative to the detection of conventional biomarkers [145]. Due to its porous nature, the paper has an inherent hydrophilic feature that is particularly useful since it may promote the flowing of fluid via capillary reaction without external pumping [146]. PADs are less expensive and simpler to make than sensors composed of materials such as glass, silicon, and plastic [147]. Additionally, PADs can be disposed of in an environmentally friendly manner and are simple to store, transport, and store. Three types of PADs include dipstick assay, lateral flow assay (LFA), and microfluidic paper-based analytical device [148]. Membranes made of cellulose and nitrocellulose are the two main forms of paper substrates.

**Figure 4:**
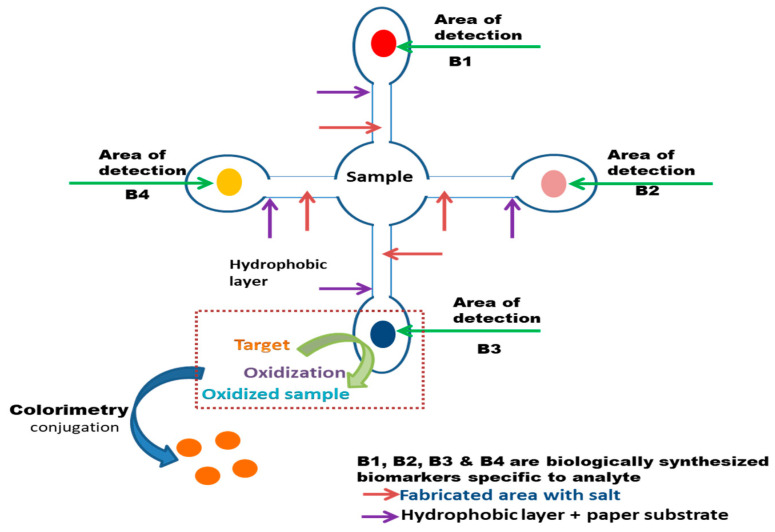
Representation of a basic paper-based biosensor via microfluid mechanism for the target.

Paper-derived colorimetric sensors are able to use enzyme-based reactions and have the ability for real-field diagnosis. The sample is put onto a test strip, and the result is displayed within 5 to 10 minutes, using the paper-based lateral flow immunochromatographic analysis test, which analyzes the quality and quantity of the material in a complicated mixture [75]. A paper-based colorimetric sensor was used to predict plant diseases in *Allium* crops for the easy and simple measurement of PG activity with the help of the principle of the ruthenium red (RR) dye method [149]; however, the drawback of hindrance can be developed by blurry signaling at the detection point due to capillary reaction forces on the liquid and its corresponding membrane [150]. Figure 4 illustrates the fundamental functioning of a microfluid paper-based biosensor, implemented with the colorimetric methodology for the observation of color change in case of any targeted toxins detection.

### 7.3. Surface Plasmon Resonance (SPR)-Based Biosensing

SPR-derived sensors are suitable for the measurement of biological molecular associations in real time, and are helpful analytical tools for calculating the adsorption rate of target analytes onto molecularly derived probes connected with a metallic surface [151]. The electromagnetic (EM) resonance of the group oscillations of free electrons connected to a plasmonic metal (silver or gold for visible spectrum) and a dielectric semi-infinite interface is known as surface plasmon resonance (SPR) [152]. This resonance produces a linked, exponentially decaying surface EM field along the metal dielectric contact. This field can be employed as a sensing layer to implement SPR-based sensors since it is particularly sensitive to changes in the refractive index (RI) of the dielectric layer [153]. The SPR-based biosensor was utilized for the detection of the fungus *P. fijiensis* in samples of banana leaf extracts [154]. However, plasmonic sensors also have many drawbacks, such as nonspecific surface binding, mass transportation restrictions, steric hindrance during the binding event, and data misinterpretation during common events [88]. Localized surface plasmon resonance (LSPR) is another promising method related to SPR biosensing with high sensitivity, a type of SPR phenomenon in which the resonant EM field is constrained to the metallic nanostructure and sensitive to RI change of the medium surrounding it only within a few tens of nm [155]. For colloidal and randomly oriented nanoparticles, scattering and absorption effects are dominant. The advantages of LSPR over SPR include a high aspect ratio that permits a larger interaction surface area for immobilizing the sensing elements, a miniaturized probe to create compact devices, and broad applicability and compatibility with a variety of phenomena, including fluorescence, Raman, and IR spectroscopy, among others less perceptive than traditional SPR [156,157].

### 7.4. Chip-Based Rapid Sensing

In chip-based biosensors, the sensing layer is created over a thin metal (50 nm)–coated glass substrate, and the analyte to be recognized flows through a microfluidic channel near the sensing layer [153]. As mentioned, these chip-based sensors have many benefits, including label-free detection, dynamic measurement of binding–unbinding kinetics to watch the reaction mechanism occurring over the sensing surface, and high sensitivity [158]. Microfluidics, for example, can be integrated with chip-based substrates that are highly sensitive, repeatable, and miniaturized [155]. Chip-based biosensors are appropriate for microfluidics-based methods, which allow mixing, filtering, and detection to be carried out on the same chip by adjusting tiny amounts of liquid. The DNA of *Ganoderma boninense*, a pathogenic fungus that infects oil palm trees in Malaysia, was extracted using a microfluidic chip device [159]. The increasing research and development in this area have demonstrated its potential for real-time evaluation and mobility, as well as prospective future works in terms of the improvement of multiple biosensors and multiple pathogen detection in a single-chip platform [159,160].

### 7.5. Electrochemical Sensing

In electrochemical-based biosensors, a molecular recognition coating and an electrochemical transducer, which transforms biological data obtained from a connecting event into an electrical signal and then displays them on a readout device, are two main components [161]. Different types and forms of nanomaterials and nanocomposites could be used in electrochemical biosensors to improve the sensitivity of the detection mechanisms and to give better detection limits through various tactics [126,162]. Microfluidic systems and electrochemical biosensors can be integrated to provide miniaturized components on a single platform [163]. The urediniospores of soybean rust fungi were detected with aptamer-based sensitive electrochemical sensing. For this, spore-bound aptamer units were incubated with a streptavidin–alkaline phosphatase agent, leading to the enzymatic formation of p-nitrophenol, which is characterized by its unique electrochemical properties [164]. Regarding the electrochemical nano-biosensor of bacterial species, recently, an electrochemical-based immune sensor was manufactured for the detection of *Escherichia coli* O157:H7 (*E. coli* O157:H7) using cyclic voltammetry (CV) with the combination of nanomaterials [143,165]. An electrochemical-derived impedance immune sensor for the detection of strained *Staphylococcus aureus* was also introduced [166].

**Figure 5:**
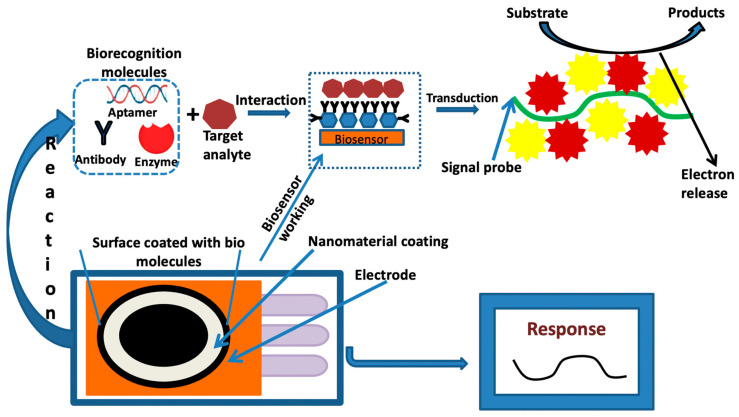
Pictorial representation of the fundamental process of the electrochemical-based nanosensor.

Electrochemical methods, such as electrochemical impedance spectroscopy (EIS), differential pulse voltammetry (DPV), cyclic voltammetry (CV), square wave voltammetry (SWV), and amperometry, have been implemented for pathogen detection and identification [167]. Nanomaterials or polymers have been normally applied with these methods to improve signals. Figure 5 defines the fundamental principle of the electrochemical-based nanosensor techniques for any kind of desired analyte.

Electrochemical-based platforms are the most successful biosensors, and they have been used for the detection and identification of huge numbers of biomarkers and the diagnosis of infectious diseases, including cancers [168]. Despite the advantages of electrochemical-based biosensors, there are a few problems associated, such as analyte detection and analyte loss during the transporting of the sample to the electrode surface [169,170]. By assessing the different applications and utility of these sensing techniques with plant pathogens, it was discovered that they are fast, sensitive, accurate, specific, and suitable for their portability. This can be applied with slight modifications to manufacture an efficient tool for the detection of *Alternaria*.

## 8. Idea for Suitable Future Strategies in the Detection of *Alternaria* Species 

In several research disciplines, such as environmental tracking, diagnostic of various airborne lethal pathogens, real-time diagnostic of contents in human blood and disease-causing microorganisms, and detection of pesticide substances in food products and beverages, biosensors have emerged as cutting-edge detection techniques [72,171,172]. To date, no suitable strategies have been reported for the detection of *Alternaria* in the field. There are only a few developed strategies that are advanced and capable of detecting *Alternaria*. For example, in pomegranate, *Alternaria* spp. cause quality issues in postharvested fruits. They infect pomegranate at an early stage of ripeness, grow, and spread within the fruit, and degrade the cell wall by developing alternariol (AOH), alternariol methyl ether (AME), and tenuazonic acid (TA) toxins and detoxifying resistance compounds in the fruit [173]. To resolve this, most analytical methods, such as nuclear magnetic resonance (NMR) and magnetic resonance imaging (MRI), have been utilized to characterize and detect the black heart but are often time-consuming and require a complicated sample pretreatment [174]. 

For the identification of *Alternaria*, researchers have used a technique such as the electronic nose (E-nose) technique [175] due to it being a fast and nondestructive technique that imitates the human olfactory system [176]. Compared with other methods [177], it presents precise and stable performance, a simple sample pretreatment procedure, a short measurement time, low cost, and ease in handling [11]. Composed of metal oxide semiconductor (MOS) biosensors, the E-nose has been utilized in detecting the changes in different food products during storage time and is helpful in the assessment of shelf life and spoilage [175,178].

A DNA-HRP functionalized silver nanoparticle probe was developed for the detection of *Alternaria* in citrus [179]. Therefore, there is a requirement of eco-friendly materials and biodegradable printable polymers with high conductivity in electrochemical biosensors and colorimetric biosensors, which leads to the identification of *Alternaria* toxins in desired samples [180]. Developing alternative methods that overcome the drawbacks of conventional methods and a lab-based method in detecting *Alternaria* spp. fungus in medicinal plants is necessary.

Current developments in micro- and nanotechnology have made it possible to create biosensor-based tests that deliver incredibly accurate, sensitive, and quick results. For general adoption and implementation in agricultural and horticultural areas, there are numerous additional factors and potential difficulties that must be taken into account [181]. Additionally, the *in-situ* sampling technique has a significant impact on the precision of biosensor-based detection [67]. To control and possibly prevent the transmission of lethal plant diseases and plant pathogens, such as *Alternaria*, to new areas in medicinal plants, it is imperative to develop a rapid, efficient, and cost-effective methodology for the early detection of disease-causing pathogens. To stop the spread of disease with the least amount of damage to crop productivity and food safety, rapid detection systems with high sensitivity and selectivity for plant pathogens are crucial. For the quick and precise identification of *Alternaria*, nanotechnology systems provide an option in which all detection procedures are carried out by a portable, miniaturized instrument [133,182]. Additionally, pathogen detection requires several desired qualities, in which functionalized metal nanoparticles as a sensing component of nanomaterials give advanced sensing capabilities for use in the field [62,66]. Over the past few decades, biosensors have been used for the detection and monitoring of a wide variety of bacterial species. These gadgets are highly sensitive detection platforms with small detection limits. Electrochemical biosensors and colorimetric biosensing among other forms of biosensors are particularly adept at avoiding many issues with the use of traditional and common laboratory methods [183].

This chapter gives a general overview of how nanotechnology is being used in the field of microbiological diagnostics, with an emphasis on medicinal plant pathogens, in particular, *Alternaria*.

## 9. Conclusions

This paper briefs and offers insights on technologies that are frequently used for detecting various diseases and pathogenic microbes in plants in the past few years and their associated problems. Advancement in the technologies is required to overcome various challenges that arise in these techniques, including the sensitivity, selectivity, and specificity in the detection system using nanotechnology. Diagnostic instruments that are conventional/traditional are quickly being replaced by nano-biosensing-based technologies and devices. These low-cost, quick, portable, very sensitive, and specialized tools for the field detection of pathogens associated with significant medicinal plants will soon find widespread adoption with further experimentation for application in a variety of environments. With the use of these portable nano-biosensors, the frequency and volume of chemically derived materials and their applications to herbs both before and after harvesting, the costs associated with on-farm production, and the loss of herbs’ quality and yield due to plant pathogen diseases would all be significantly decreased. However, more research is needed to improve these nano-biosensors so they can simultaneously monitor and detect many disease-causing fungi/bacteria on the field. Electrochemical-based wearable biosensors with nanofabrication of biomolecules on suitable nanomaterials have the ability to sense wirelessly, detect, and give signals of the presence and abundance of pathogenic organisms and, therefore, can be used for the early detection of *Alternaria* strains in medicinal plants that will also improve their production and quality. In this review, the applications of a variety of sensors as a diagnostic tool were highlighted. These tools typically combine a biological sensing component with a physicochemical transducer, electrochemical properties, and colorimetric-based technology. These tools can provide the desired identification strategies for the *Alternaria* disease detection in medicinal plants and help to improve early detection and enable the most effective prevention for farmers and the economy.

## Figures and Tables

**Table 1:** Different fungal diseases in medicinal plants.

Disease	Strain	Family	Infection in Medicinal Plants	Region	References
Black spot	*A. alternata*	*Pleosporaceae*	*Paris polyphylla* var. chinensis	China	[24]
Leaf spot	*A. alternata*	*Pleosporaceae*	Adosa (L.) Nees	Bangalore	[4]
Leaf spot	*A. alternata*	*Pleosporaceae*	Safed musli (*Chlorophytum borivilianum*)	Karnataka	[25]
Leaf blight	*A. alternata*	*Pleosporaceae*	Noni (*Morinda citrifolia* L.)	Tamil Nadu and Karnataka	[26]
Leaf blight	*A. alternata*	*Pleosporaceae*	Tulsi (*Ocimum sanctum* L.)	West Bengal	[27]
Leaf spot	*A. brassicae*	*Pleosporaceae*	Aloe vera (*Aloe barbadensis* Mill.)	Parganas, West Bengal	[28]
Leaf blight	*A. tenuis*	*Pleosporaceae*	Sarpagandha (*Rauwolfia serpentina* (L.) Benth. Ex Kurz)	Bengaluru	[29]
Leaf blight	*A. alternata*	*Pleosporaceae*	African basil (*Ocimum gratissimum* L.)	Karnataka	[30]
Leaf spot	*A. alternata*	*Pleosporaceae*	Mint (*Mentha arvensis* L.)	Jammu and Kashmir	[31]
Leaf blight	*A. alternata*	*Pleosporaceae*	*Catharanthus roseus* L. G. Don.	Haryana	[32]
Leaf spot	*A. tenuis*	*Pleosporaceae*	Ashwagandha (*Withania somnifera*)	West Bengal	[33]
Leaf blight	*A. alternata*	*Pleosporaceae*	Senna (*Cassia angustifolia*)	Himachal Pradesh	[34]
Leaf spot	*Colletotrichum gloeosporioides*	*Glomerellaceae*	Ashwagandha (*Withania somnifera*)	West Bengal	[35]
Leaf spot	*Xanthomonas campestris*	*Xanthomonadaceae*	Giloy (*Tinospora cordifolia* (Thunb.) Miers)	Karnataka	[36]
Basal stem rot	*Fusarium oxysporum*	*Nectriaceae*	Aloe vera (*Aloe barbadensis* Mill.)	Bali	[37]
Damping off	*Phytophthora parasitica* and *Rhizoctinia solani*	*Peronosporaceae* and *Ceratobasidiaceae*	Belladonna (*Atropa belladonna* L.)	Jodhpur	[38]
Rust	*Puccinia asparagi*	*Pucciniaceae*	Asparagus (*Asparagus officinalis* L.)	Michigan State	[39]
Wart	*Synchytrium lepidagathidis*	*Synchytriaceae*	Kalmegh (*Andrographis paniculata* (Burm. f.) Wall. ex Nees)	West Bengal	[40]
Damping off	*Rhizoctonia solani*	*Ceratobasidiaceae*	Ashwagandha (*Withania somnifera*)	Udaipur	[41]
Sclerotinia blight	*Sclerotinia sclerotiorum*	*Sclerotiniaceae*	Mint (*Mentha arvensis* L.)	Jammu and Kashmir	[31,42]
Wilt	*Fusarium solani*	*Nectriaceae*	Ashwagandha (*Withania somnifera*)	Lucknow	[33]

## Data Availability

Not applicable.
